# Does the Precision of a Biological Clock Depend upon Its Period? Effects of the Duper and *tau* Mutations in Syrian Hamsters

**DOI:** 10.1371/journal.pone.0036119

**Published:** 2012-05-15

**Authors:** Eric L. Bittman

**Affiliations:** 1 Department of Biology, University of Massachusetts, Amherst, Massachusetts, United States of America; 2 Program in Neuroscience and Behavior, University of Massachusetts, Amherst, Massachusetts, United States of America; Vanderbilt University, United States of America

## Abstract

Mutations which alter the feedback loops that generate circadian rhythms may provide insight into their insensitivity to perturbation robustness) and their consistency of period (precision). I examined relationships between endogenous period, activity and rest (τ_DD_, α and ρ) in Syrian hamsters using two different mutations, duper and *tau*, both of which speed up the circadian clock. I generated 8 strains of hamsters that are homozygous or heterozygous for the *tau*, duper, and wild type alleles in all combinations. The endogenous period of activity onsets among these strains ranged from 17.94+0.04 to 24.13±0.04 h. Contrary to predictions, the variability of period was unrelated to its absolute value: all strains showed similar variability of τ_DD_ when activity onsets and acrophase were used as phase markers. The τ_DD_ of activity offsets was more variable than onsets but also differed little between genotypes. Cycle variation and precision were not correlated with τ_DD_ within any strain, and only weakly correlated when all strains are considered together. Only in animals homozygous for both mutations (super duper hamsters) were cycle variation and precision reduced. Rhythm amplitude differed between strains and was positively correlated with τ_DD_ and precision. All genotypes showed negative correlations between α and ρ. This confirms the expectation that deviations in the duration of subjective day and night should offset one another in order to conserve circadian period, even though homeostatic maintenance of energy reserves predicts that longer intervals of activity or rest would be followed by longer durations of rest or activity. Females consistently showed greater variability of the period of activity onset and acrophase, and of α, but variability of the period of offset differed between sexes only in super duper hamsters. Despite the differences between genotypes in τ_DD_, ρ was consistently more strongly correlated with the preceding than the succeeding α.

## Introduction

The physiology and behavior of multicellular organisms is coordinated by remarkably precise circadian pacemakers whose genetically determined endogenous period (τ)remains close to 24 h in constant conditions. In nature, daily cues entrain circadian oscillations of plants and animals: zeitgebers elicit phase advances or delays in order to reset the period of the biological oscillator to exactly 24 h. It has been suggested that there is an adaptive advantage to setting τ at a value different than 24 h, as this insures that the process of entrainment will cause the organism to adopt a unique phase angle with respect to the LD cycle [1]. On the other hand, variability of endogenous period is likely to have negative selective consequences as it may result in differences within and between individuals in their pattern of entrainment. Comparisons of circadian function among four species of rodents led Pittendrigh and Daan [2] to propose that τ is less variable the closer its value is to 24 h. They offered as an ultimate explanation of this trend the idea that stability of the entrained phase angle would be more adversely affected were the clock to run erratically. Aschoff et al. [3] proposed that the cycle-to-cycle deviations in the duration of the subjective day and night compensate for one another in order to maintain constancy of period. They devised a model in which rest and activity are controlled by a circadian oscillation of arousal which passes a threshold twice each cycle. This led to a variety of predictions about the relationships between the variability of and relationship between the values of activity time (α), rest time (ρ), and τ which they tested in birds and humans. Aschoff et al. [3] presented evidence for minimum variability at particular values of τ, and found (1) a negative correlation between α and the duration of both the preceding and the following ρ; (2) dependence on τ of the correlation between α and the preceding or the following ρ and (3) dependence of the variability of τ_onset_ vs. τ_offset_ on the mean period. They also predicted a compensatory mechanism that insures that (4) standard deviation of τ is smaller than the summed standard deviations of α and ρ.

Our understanding of the molecular basis of circadian oscillations has grown dramatically since these classical formal studies were done, and insight into the means by which their robustness is insured has consequently increased [4], [5]. In a variety of organisms, transcriptional-translational feedback loops are critical to generation of these rhythms, and post-translational modifications of clock proteins may determine their period. While natural selection presumably acts in the wild upon mutations that affect these molecular processes, laboratory studies can provide insight into the consequences of such mutations for the variability and precision of circadian rhythms.

The first mammal circadian period mutant to be discovered was the *tau* hamster [6]. The period of free running locomotor rhythms in constant darkness (τ_DD_) of *tau* heterozygotes (whose genotype is here designated Tt) was 22 h, and crosses between such animals generated wild type (TT), heterozygous, and homozygous (tt) mutants in a 1∶2∶1 Mendelian ratio. In tt hamsters, described colloquially as “super short,” τ_DD_ is reduced to 20 h. The *tau* mutation is an allele of *casein kinase 1ε* which results in a gain of function, causing hyperphosphorylation of the PERIOD2 protein and abbreviation of its nuclear residence time [7]–[9]. In the course of experiments in our laboratory, a new mutation affecting circadian period arose spontaneously on the tt background. As described elsewhere [10], [11], τ_DD_ of such hamsters is approximately 18 h, and we refer to these mutants as “super duper.” Appropriate crosses generated hamsters expressing the duper mutation on a wild type background. Such animals, genotypically designated here as TTdd, show a shortening of τ_DD_ by slightly over an hour relative to the wild type. Thus the magnitude of the effect of the duper mutation on τ_DD_ is comparable to that of mouse mutants and knockouts that have proven useful in discovery of key molecular components of the circadian mechanism (see [12] for review). Although the genetic basis of the duper mutation is as yet unknown, we have established that duper hamsters differ from *tau* mutants in that (1) the mutation is recessive, (2) the coding sequence of CK1*ε* (as well as CK1δ does not differ from wild type [10], and (3) phase shifting responses to light are markedly amplified within 2 days of transfer to DD [11].

The present experiments were performed on hamsters that were homozygous or heterozygous for *duper* on either the wild type or *tau* mutant homozygote or heterozygote backgrounds. I generated a variety of strains in order to test generalizations and predictions about formal properties of circadian rhythms [1]–[3] over a broad range of periods while avoiding the use of multiple species or light intensities. This allowed me to examine the idea that variability is minimal at particular values of τ, and to test whether mutations which alter τ necessarily degrade precision. I also compared the effects of the *tau* and duper mutations on circadian period with their influence on α and ρ in order to ask whether shortening of a particular circadian phase contributes disproportionately to the reduction in period length.

## Materials and Methods

### Animal Maintenance

Syrian hamsters (LVG strain, originating from the Lakeview hamstery, Billerica MA) were born and raised in 14L:10D. They were allowed *ad libitum* access to food and water throughout the experiments. During the light phase, white fluorescent bulbs provided approximately 400 lux at cage level. As adults (mean age 97.2 d) hamsters were transferred to individual plastic tubs containing running wheels (17 cm diameter) and placed in DD, where locomotor activity was continuously monitored by computer and analyzed in 10 min bins. All procedures were approved by the animal care and use committee (IACUC) of the University of Massachusetts at Amherst, and conform to all USA federal animal welfare requirements.

### Assessment of Genotype and Circadian Phenotype

Circadian rhythms of locomotor activity were assessed using Actimetrics software as previously described [10], [11]. Because different measures of period have been reported to vary in their variability, I examined not only the period (τ_DD_) of running onsets, but also that of the acrophase of activity, and of running offsets. Unless otherwise specified, τ_DD_ estimates were based on the least square regression line fitted to the corresponding data points during each animal’s free run in DD. Phase variation of locomotor activity markers, as defined by Daan and Oklejewicz [13], was assessed using the mean standard deviation of the time of activity onsets, offsets, or acrophase around this regression line. α was assessed by direct measurement of the intervals between successive onsets and offsets of activity, and ρ as the intervals between successive offsets and onsets, over 10–12 cycles, and by linear regression fits of onsets and offsets generated by Actimetrics software using the supplier’s default settings. This software also quantified the mean number of revolutions per cycle. Cycle variation was calculated as the standard deviation of 10 successive periods based on activity onsets in DD in wild type and mutant hamsters. In order to express the variability relative to the mean value of τ_DD_, precision was determined as described by Aschoff et al. [3] as the quotient generated by dividing the mean period of successive activity onsets by the standard deviation of this measure, i.e., the inverse of the coefficient of variation.

Super duper mutants were identified and the duper mutation was isolated on a wild type background through backcrosses as previously described [10]. Briefly, skin samples (ear clips) were enzymatically digested to obtain genomic DNA and CK1ε was amplified as described by Lowrey et al. [7]. Restriction digestion was carried out using *BstAPI* in order to identify the *tau* locus, which results in a 137 bp cleavage product that can be visualized on a Metaphor gel. Animals resulting from the F_2_ cross of wild type X super dupers that lacked the *tau* mutation (i.e., carry only wild type CK1ε) but whose τ_DD_ was below 23.2 h were founders of the duper line. Crosses of these duper hamsters invariably produce offspring with 22.7<τ_DD_ <23.2 h. I also crossed duper homozygotes that lacked the CK1ε mutation with wild type hamsters in order to produce hamsters that were heterozygous for the *duper* allele but bore no other circadian mutations (TTDd). In addition I crossed duper homozygotes with ttDD hamsters in order to produce offspring that were heterozygous for both *duper* and the *tau* alleles (TtDd). I also assessed circadian rhythms of F_2_ offspring of duper hamsters that were heterozygous for the *tau* (CK1ε) mutation (here designated Ttdd).

### Statistical Evaluations

The amplitude of the wheel running rhythm over a 10-day interval in DD was assessed by three methods: the same raw data were subjected to analysis by the Chi square periodogram, which provides an estimate based on the Sokolove-Bushell method [14], the Fast Fourier transform, and autocorrelation. Analyses were performed using JMP Statistical software (version 8.0.2, SAS Institue Inc., Cary, NC) for 1-way ANOVA and regression analysis to evaluate effects of genotype on cycle variation and precision, and 2-way ANOVA to evaluate main effects of genotype and sex and their interaction. Post-hoc comparisons were performed using Tukey-Kramer tests as appropriate. Student’s t-test was performed for pair-wise tests. Microsoft Excel and JMP Statistical software were used to perform linear and polynomial regressions and to calculate correlation coefficients between α, ρ and τ_DD_ and evaluate their statistical significance.

## Results

### Influence of *Tau* and Duper Alleles on Circadian Period and its Variability

When hamsters of both sexes were considered together, τ_DD_ differed markedly among the 8 strains regardless of whether the onset or offset of activity or the acrophase was used as a phase marker (*P*<0.0001; Table 1). Each of the mutant strains differed significantly from the wild type in the period of activity onset (*P*<0.0001) with the single exception of the duper heterozygotes (TTDd, confirming that the duper mutation is recessive). Sex had no significant influence on τ_DD_ regardless of which phase marker was used, but there was a significant interaction between genotype and sex when either onset or acrophase was used to determine τ_DD_ (*P*<0.03).

**Table 1:** Characteristics of circadian rhythms in wild type and mutant hamsters of both sexes.

	Wild type (TTDD)	τ_s_ (TtDD)	τ_ss_ (ttDD)	Duper (wt bkg; TTdd)	Super duper (τ_ss_ bkg; ttdd)	wt/het duper (TTDd)	τ_s_/het duper (TtDd)	τ_s_/homo zygous duper (Ttdd)
Period of activity onsets (τ_DD_)	24.06±0.02h^a^ n = 92	22.38±0.06h^c^ n = 50	20.11±0.06h^d^ n = 46	22.82±0.05h^b^ n = 101	17.94±0.04h^e^ n = 64	24.13±0.04h^a^ n = 25	22.39±0.05h^c^ n = 15	19.87±0.05h^d^ n = 37
Variability of activity onsets	0.568±0.058h^a,b^	0.566±0.083^a,b^	0.564±0.050^a,b^	0.435±0.034^b^	0.596±0.044^a,b^	0.758±0.075h^a^	0.558±0.125^a,b^	0.590±0.047^a,b^
Period of activity offsets	24.02±0.02h^a^	22.30±0.06h^c^	20.08±0.06h^d^	22.81±0.06h^b^	17.95±0.04h^e^	24.02±0.04h^a^	22.27±0.07h^c^	19.88±0.06h^d^
Variability of activity offsets	1.35±0.05h^a^	1.21±0.07h^a^	1.15±0.06h^a^	1.47±0.05h^a^	2.12±0.81h^a^	1.70±0.16h^a^	1.22±0.22h^a^	1.16±0.06h^a^
Period of acrophase	24.06±0.02h^a^	22.35±0.06h^c^	20.10±0.06h^d^	22.82±0.05h^b^	17.93±0.04h^e^	24.10±0.02h^a^	22.31±0.05h^c^	19.95±0.09h^d^
Variability of acrophase	0.586±0.038h^a,b^	0.575±0.043h^a,b^	0.719±0.071h^a^	0.507±0.033h^b^	0.711±0.037h^a^	0.609±0.058h^a,b^	0.671±0.151h^a,b^	0.695±0.057h^a,b^
Activity duration (α)	7.36±0.13h^a,b^	6.28±0.22h^c,d^	5.73±0.15h^d^	6.95±0.12h^b,c^	4.94±0.16h^e^	8.13±0.26h^a^	5.58±0.33h^d,e^	6.26±0.19h^c,d^
# wheel revs/cycle	6099±306^b^	4053±372^c,d^	2744±293^d,e^	7503±264^a^	7165±457^a,b^	1885±170^e^	8015±484^a,b^	4594±336^c^

a, b, c, d: common superscripts within each measure indicate genotypes do not differ significantly (p>0.05, Tukey’s HSD). Variability of activity onsets, offsets, and acrophase refers to the mean of the standard errors of the fit of the regression line used to estimate free running period in individual hamsters of each of the indicated genotypes.

Despite these striking differences between these eight hamster strains in τ_DD_, the *phase variation* (assessed as mean of the standard error of fit to the regression line of the activity rhythm) did not differ when activity onsets or acrophase were used as phase markers (Table 1). Sex had a significant main effect on phase variation, with females showing less regular activity onsets or acrophases (*P* <0.0002). There was no interaction between genotype and sex on the variability of activity onset or acrophase. There were significant main effects of genotype (*P*<0.0001) and sex (*P* = 0.01) on the variability of activity offsets, as well as a significant interaction between genotype and sex (*P*<0.0001).

In order to assess further the relationship between the period of the oscillation and its variability, we calculated both the *cycle variation* and the *precision* in different hamster strains over 10 successive cycles. The former provides a measure of the raw variability of cycle length, while the latter normalizes this variability to circadian period and represents the reciprocal of the coefficient of variation [3]. When hamsters of both sexes were consider together, cycle variation did not differ with either genotype or sex, and there was no significant interaction between these factors. In contrast, the precision of free running rhythms (estimated from τ_DD_/sd based on activity onsets) differed significantly with genotype (*P*<0.001): precision was significantly higher in TTdd and TtDD than in ttdd hamsters (p<0.05). Other groups did not differ significantly from one another in precision, which was intermediate between these extremes (including in wild type hamsters; Tukey’s test). Precision was also significantly higher in males than females (*P*<0.0001), but the interaction between genotype and sex was not statistically significant (*P* = 0.08). In light of the difference between sexes in measures of phase and cycle variation which may result from fluctuations in ovarian hormone levels, further analyses were restricted to male hamsters.

**Figure 1:**
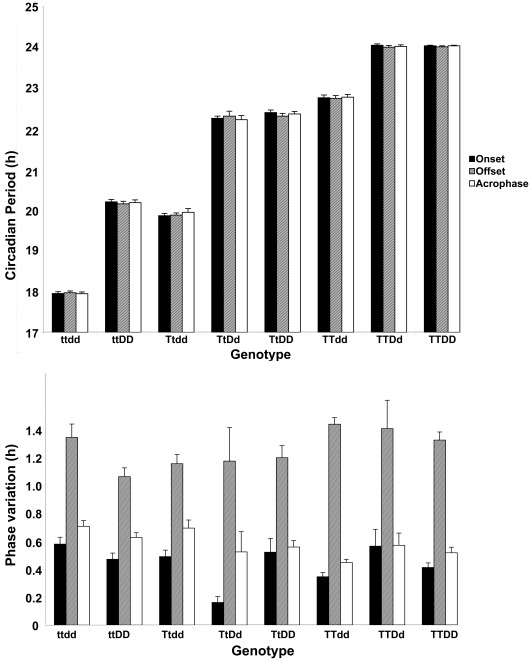
Effect of genotype on circadian period and its variability in male hamsters. Mean (±SEM) τ_DD_ (top) and phase variation (bottom) of the time of activity onset (black bars), offset (hatched bars) or acrophase (white bars) of the first-order regression line used to determine τ_DD_ in male hamsters bearing wild type, *tau*, and duper alleles in various combinations. Posthoc analysis indicated statistically significant differences in τ_DD_ of activity onsets at the *P*<0.05 level: TTDd = TTDD>TTdd>TtDD>ttDD>Ttdd>ttdd (Tukey HSD test; TtDd did not differ from TTdd and TtDD). Statistical differences between genotypes in τ_DD_ of activity offset and acrophase showed similar patterns. Note that while strains differ markedly in τ_DD_, phase variation is generally similar between genotypes. For phase rariation of activity onset and offset, only ttdd and TTdd groups differed from each other at the *P*<0.05 level; for phase variation of acrophase, ttdd>TTDD = TTdd. In all genotypes, phase variation of τ_DD_ based on activity offset is greater than phase variation of τ_DD_ assessed for onsets or acrophase.

Both τ_DD_ and phase variation differed among males of the various genotypes regardless of whether the onset, offset, or acrophase of activity was used as phase marker (*P<*0.003; Fig. 1). Nevertheless, phase variation of male hamsters was not consistently related to circadian period, and only ttdd and TTdd males differed significantly from one another in the error of the fit of the linear regression used to determine τ_DD_. In order to normalize the phase variation to circadian period, the standard error of the fit of the linear regression to the activity onsets was divided by τ_DD_. When the variability of onsets is considered as a proportion of τ_DD_ in this way, there was a significant effect of genotype (*P*<0.0001). Genotypes with the shortest mean τ_DD_ tended to have the least precise onsets. Thus male TTDD and TTdd hamsters showed significantly less scatter in the fit of the regression line relative to the period of activity onsets than did ttDD or ttdd hamsters (*P*<0.05), but other genotypes did not differ. Across all genotypes, both the linear and quadratic regression fits were significant (R^2^ <0.14, *P*<0.0001). Nevertheless, there is a wide range of variability even among TTDD and TTdd hamsters, and neither the linear nor the quadratic regression fit of the relationship between the error of activity onsets and τ_DD_ reached significance within any genotype.

**Figure 2:**
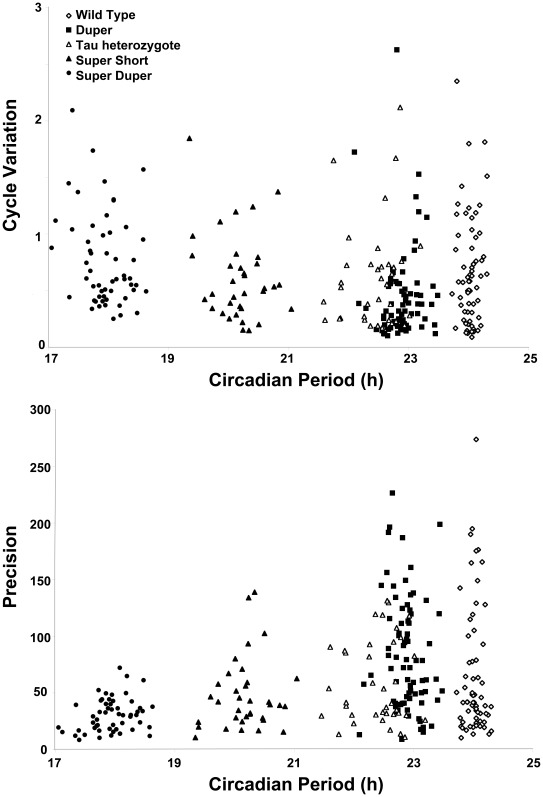
Relationship between τ_DD_, cycle variation and precision in male Syrian hamsters. (A) Scatter diagram depicting standard deviation (in hours) of the mean period of successive activity onsets in individual hamsters. (B) precision (standard deviation normalized to period) in TTDD (diamonds), TTdd (squares), ttDD (filled triangles), TtDD (open triangles), and ttdd hamsters (circles) in the same individuals shown in (A). Neither a linear nor a quadratic fit provides evidence for a minimum cycle variation at a particular τ_DD_ value within any of these four genotypes (R^2^<0.3; *P* >0.08). When all genotypes are combined, a weak quadratic trend is statistically significant for both standard deviation (R^2^ = 0.14, *P* = 0.02) and precision (R^2^ = 0.14, *P* = 0.03).

**Figure 3:**
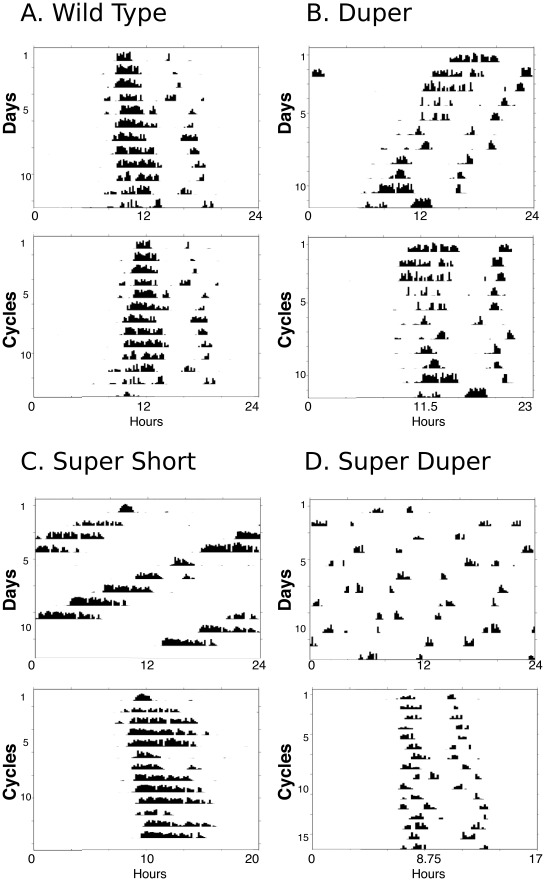
All strains show coherent rhythmicity despite differences in precision. Actograms depicting locomotor activity rhythms in DD in the individuals showing the median value of circadian precision among groups of (A) TTDD, (B)TTdd, (C) ttDD, and (D) ttdd male hamsters. In each panel the top actogram is plotted modulo 24h. The same data are replotted in the bottom actogram modulo τ_DD_.

The cycle variation and precision of τ_DD_ differed significantly among males of the 8 genotypes (<0.01). Post-hoc comparisons indicated that the standard deviation of cycle length was greater in ttdd than in TTdd hamsters (*P*<0.05), but other genoytpes did not differ significantly from one another (Fig. 2). Across all genotypes, both the linear and quadratic regressions of circadian period vs. standard deviation were statistically significant (*P*<0.02) despite the low R^2^ value (<0.05). The linear regression was significant (*P*<0.05) in TTDD, TTdd, Ttdd, and ttdd hamsters (R^2^<0.17). Quadratic trends were significant in TTdd, ttDD, ttdd, TTDd and Ttdd hamsters, although the relationship between τ_DD_ and its standard deviation was strong (R^2^ = 0.74) only in the latter group (all others R^2^ <0.2). Precision, which represents variation relative to period of cycle-to-cycle onsets, also differed with genotype (*P*<0.0001; R^2^ = 0.156). This was due exclusively to super duper hamsters: precision was lower (*P*<0.05) in ttdd than in all other groups except ttDD, but was not systematically correlated with τ_DD_ (Table 2). It is important to note than though lower than in other genotypes, precision in the ttdd hamsters was adequate to insure regularity of free running rhythms (Fig. 3). Across all genotypes of male hamsters, both the linear and the quadratic regressions of precision vs. period were significant (*P*<0.0001; R^2^ <0.12; Table 2). Within males of the 8 genotypes, the linear relationship was significant only in ttdd and Ttdd hamsters, and the quadratic relationship was significant in ttdd and Ttdd hamsters, although correlation coefficients were uniformly low (Table 2; Figure S1). In no case was the precision greatest at or near the mean τ_DD_ of the genotype.

**Table 2:** Mean (± SEM) period of activity onsets and precision, and linear and quadratic regression analysis in male wild type and mutant Syrian hamsters.

genotype	n	τ_onset_	Precision	LinearR^2^	Linea*rP*	Quadratic R^2^	Quadratic *P*
TTDD (wild type)	66	24.05^a^±0.04	63.26^ab^±5.08	0.0007	0.048	.062	0.13
TTdd (duper)	80	22.87^b^±0.03	82.39^a^±4.62	0.0155	0.27	0.024	0.38
TtDD (tau heterozygote)	39	22.40^c^±0.0	58.80^ab^±6.70	0.0143	0.47	0.029	0.59
ttDD (super short)	36	20.21^d^±0.05	48.73^bc^±6.89	0.024	0.37	0.10	0.16
Ttdd	31	19.85^c^±0.52	51.94^a^±7.42	0.173	0.02	0.206	0.04
Ttdd (super duper)	55	17.96^f^±0.04	30.17^c^±5.57	0.074	0.045	0.111	0.046

Groups with different superscripts differ significantly at *P*<0.05 level. Linear and quadratic R^2^ values reflect first and second order regression fits of τ_DD_ vs. precision within the respective group, respectively. Even in cases in which regression fit was statistically significant, the maximum precision did not occur at the mean τ_DD_. When all male hamsters were combined and analyzed across genotypes, linear and quadratic fits were both statistically significant at the *P*<0.0001 level, but the R^2^ value was low (0.09 and 0.118, respectively).

### Influence of Tau and Duper Alleles on Locomotor Rhythm Amplitude and Activity Levels

The effects of genotype on the amplitude of the principal significant (circadian) component were evaluated using Chi square periodogram, FFT, and autocorrelation.

Each of these methods indicated a significant overall effect of genotype (*P*<0.001) and a significant correlation between amplitude and period (R^2^ = 0.2; *P*<0.01). Regardless of which measure was calculated, the amplitude of circadian free runs in TTDD and TTdd hamsters did not differ from each other, nor did amplitude differ between TtDD, ttDD, and ttdd hamsters. However, the amplitudes of rhythms of the former groups significantly exceeded that of the latter (*P*<0.05). Across all genotypes, both precision and τ_DD_ were significantly correlated with periodogram amplitude, FFT power, and autocorrelation coefficient (linear and quadratic fits both *P*<0.0001). The correlation between amplitude and τ_DD_ was not statistically significant within individual genotypes. The correlation of precision with amplitude was statistically significant within TTDD, TTdd, TtDD, and ttdd male hamsters.

Genotype significantly affected the mean number of wheel revolutions (*P*<0.0001), but sex did not and there was no interaction between sex and genotype. The mean number of wheel revolutions was correlated with τ_DD_ across all genotypes (*P*<0.0001). TtDd and TTdd hamsters were the most active, while ttDD and ttdd animals had the fewest wheel revolutions per cycle. Ttdd and TtDD animals were intermediate in this measure and differed significantly from both extremes (*P*<0.0001). Nevertheless, there was no correlation between the number of revolutions and τ_DD_ within TTDD, TTdd, ttDD or ttdd (R^2^<0.03; *P*>0.10).

### Influence of *Tau* and Duper Alleles on Activity and Rest

The effects of the *tau* or duper mutations on circadian period might reflect a change in the duration of a particular phase of the circadian oscillation. For example, these mutations could disproportionately alter the duration of the active period or the rest period. As pointed out by Aschoff et al. [3], deviations in α and ρ must be balanced if free running period is to be conserved. Estimates of α generated by linear regression fits of activity onsets and offsets were used to evaluate these ideas in all 8 hamster strains.

**Figure 4:**
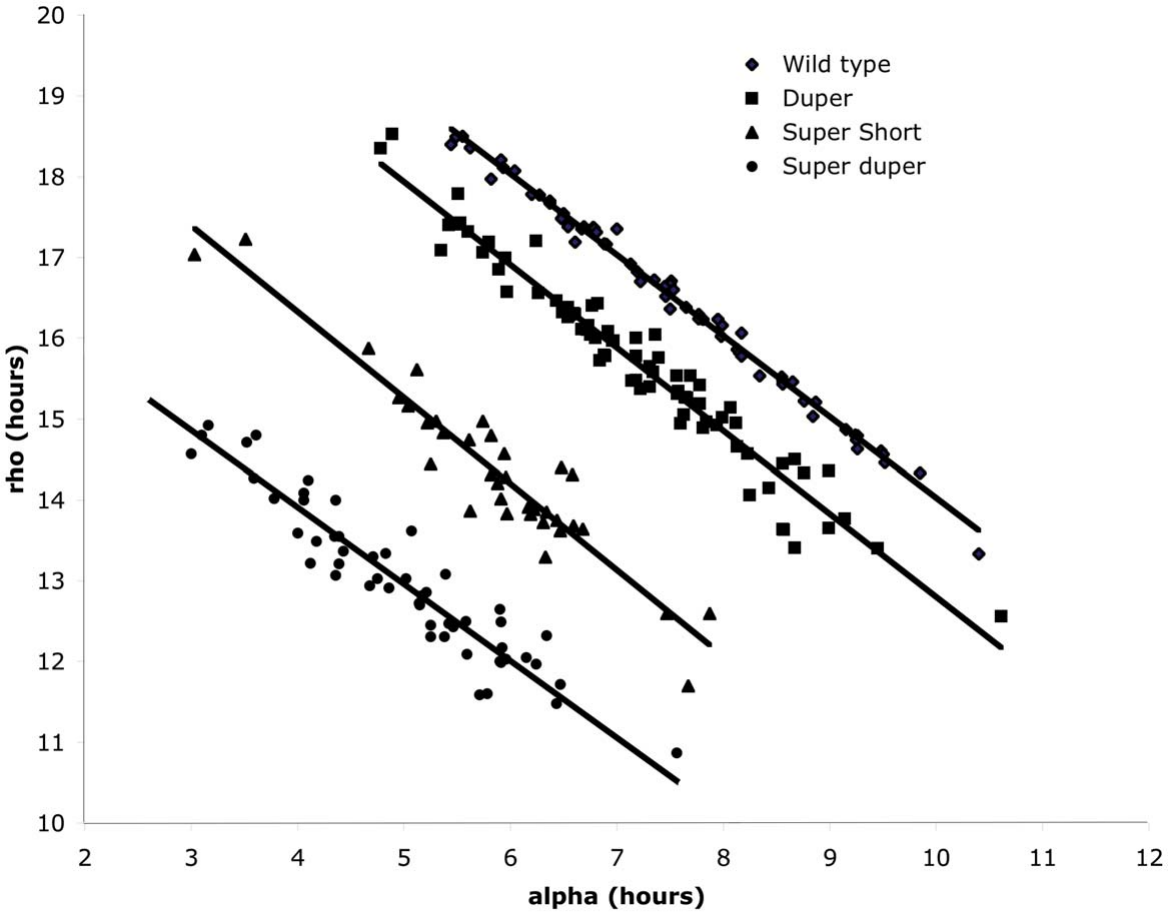
Activity and rest are negatively correlated. α and ρ are illustrated in TTDD (diamonds), TTdd (squares), ttDD (triangles), and ttdd (circles) hamsters. Compensatory and offsetting changes occur in activity and rest in order to achieve conservation of τ_DD_, contrary to predictions on the basis of energetic considerations that longer activity might lead to longer rest and *vice versa*. The relationship between activity time and rest time is similar (R^2^ >0.9) in all strains, and shortening of period in mutant hamsters reflects equally the reductions in α and ρ. Linear regressions and correleation coefficients for the four strains are as follows: TTDD: y = −1.0013×+24.042; R^2^ = 0.992; TTdd: y = −1.0271×+23.703; R^2^ = 0.9501 ttDD: y = −1.0637×+20.581; R^2^ = 0.8947 ttdd: y = −0.9511×+17.716; R^2^ = 0.9329.

**Figure 5:**
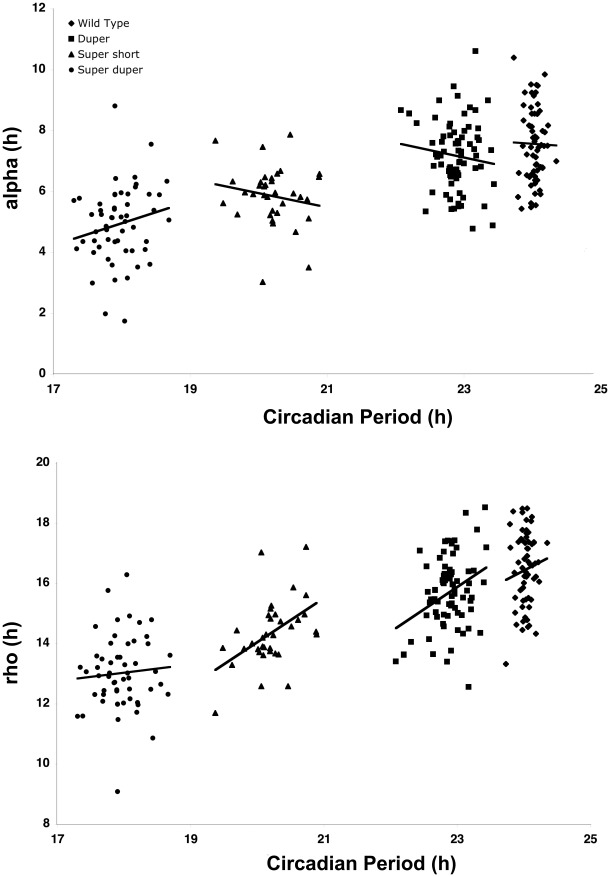
Relationships between activity or rest duration and circadian period differ between genotypes. Correlations between the period of activity onset (τ_DD_) and α (top) or ρ (bottom) in TTDD (diamonds), TTdd (squares), ?tDD (triangles), and ttdd (circles) Syrian hamsters. Both α and ρ correlated with τ_DD_ across all genotypes (linear and quadratic fits both *P*<0.0001) but in none of the individual strains was the fit of the linear or quadratic regression statistically significant.

**Figure 6:**
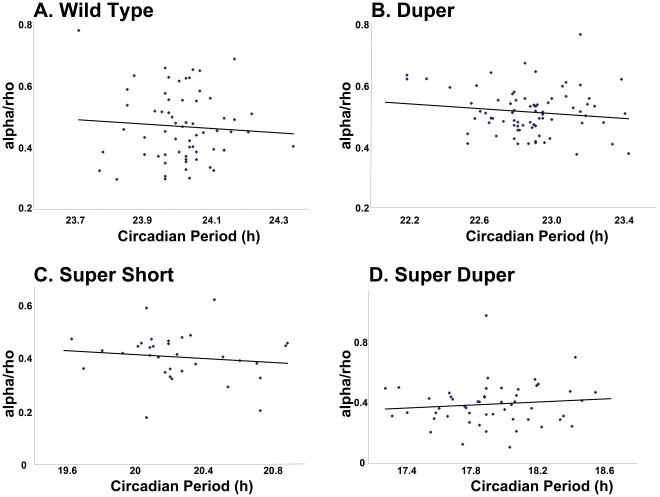
Circadian period does not predict activity:rest ratio. The relationship between the activity/rest ratio and the period of activity onsets (τ_onset_) in constant darkness was similar in TTDD, TTdd, ttDD and ttdd hamsters.

When both males and females were considered together, genotype had a significant influence on both α and the mean number of wheel revolutions per cycle (*P*<0.0001). Sex had a significant main effect on α (*P*<0.02), but there was no interaction between genotype and sex. As expected, α was correlated with τ_DD_ in male hamsters: it was greatest in TTDD and TTdd hamsters, which did not differ from each other, and least in ttdd hamsters. TtDD and ttDD hamsters had intermediate values of α and differed significantly from these extremes. When all hamsters of the various strains were considered together, τ_DD_ of activity onsets was positively correlated with α (*P*<0.001; R^2^ = 0.34). Also as expected, the effects of genotype on the number of wheel revolutions showed patterns similar to the effects on α. Similar patterns were evident when cycle-to-cycle estimates of activity onset and offset rather than regression line fits (Actimetrics software) were used to assess in 10 randomly selected male TTDD, TTdd, ttDD and ttdd hamsters. An interesting difference between the effects of the two mutations on α and the number of wheel revolutions per cycle was noteworthy: although τ_DD_ was similar in TtDD and ttDD hamsters, the decrease in activity relative to wild types evident in the *tau* mutants did not occur in the duper hamsters (Table 1). Furthermore, the number of wheel revolutions in ttdd hamsters was markedly greater than that in ttDD animals and comparable to that of wild types, indicating that the *duper* allele may exert a protective effect against the reduction in activity caused by the *tau* mutation. Confirming observations made previously in wild type hamsters, α and ρ were negatively correlated in all strains (*P*<0.002; Fig. 4). Neither α nor ρ (Fig. 5), nor the α?ρ ratio (Fig. 6) was significantly correlated with τ_DD_ when cycle-to-cycle estimates were analyzed in TTDD, TTdd, ttDD or ttdd hamsters. The α?ρ ratio was significantly reduced only in ttDD hamsters relative to wild types. There was no effect of genotype on the coefficient of variation of ρ, but the coefficient of variation of α was greater in ttdd than in TTDD and TTdd hamsters.

**Figure 7:**
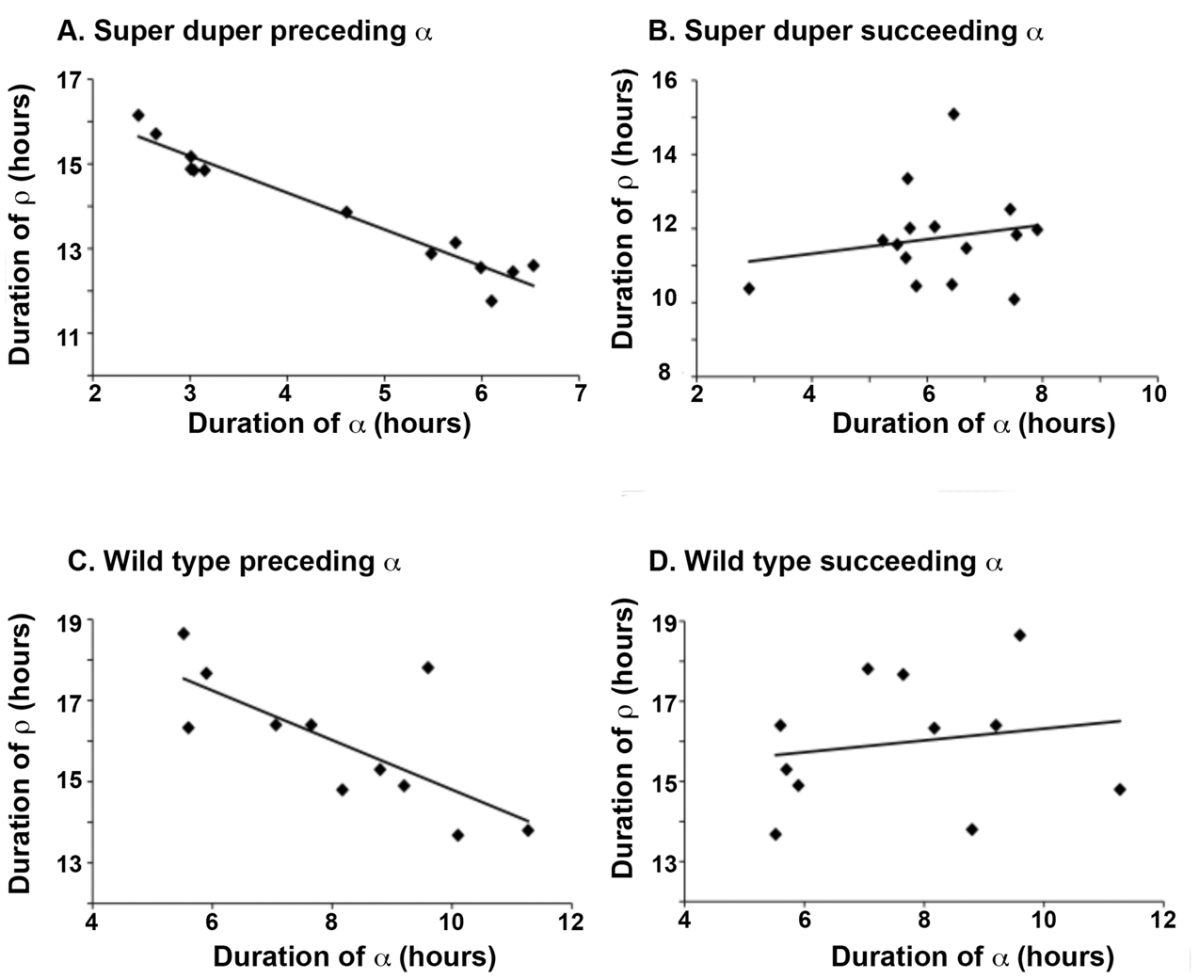
Activity duration predicts duration of the following rest period. Examination of the correlation between rest time (ρ) and the preceding (left) or succeeding (right) activity phase (α) over 10 successive circadian cycles in representative free running super duper mutant (top) or wild type (bottom) male Syrian hamsters. For the group of wild type hamsters, the correlation (R^2^) of α with the following ρ was 0.74±0.01 (n = 10, mean+SEM). In contrast, the correlation with the preceding ρ was 0.08±0.04. Among 10 super duper hamsters, the correlation of α with following ρwas 0.65±0.11, while the correlation with the preceding ρwas 0.04±0.02. Contrary to the model of Aschoff et al. [3], τ_DD_ does not predict the strength of the serial correlation.

The correlation between the duration of α *preceding* ρ was consistently greater than the correlation between ρ and the *succeeding* α (Fig. 7). This was the case for both TTDD (R^2^ = 0.744±0.10 vs. 0.085±0.037, n = 10) and ttdd (R^2^ = 0.645±0.11 vs. 0.044±0.023, n = 10) hamsters. Thus a longer activity interval predicts a shorter ensuing rest period, but a longer rest interval does not predict the length of the next active period. Contrary to predictions of Aschoff’s model [3], this pattern was independent of circadian period.

**Figure 8:**
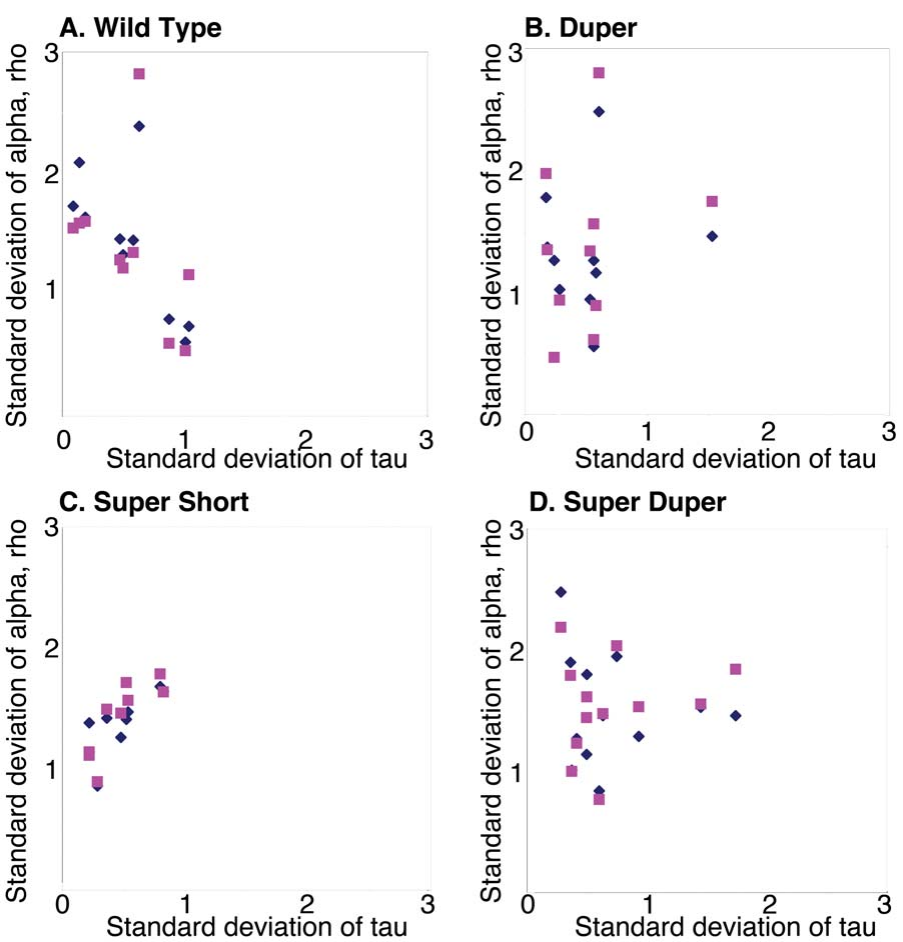
Activity and rest variability exceeds period variability. Relationship between the variability of α (diamonds) or ρ (squares) and the variability of τ_DD_ in (A) TTDD, (B)TTdd, (C)ttDD, and (D) ttdd Syrian hamsters. Data from ten randomly selected males of each genotype are represented. Note that standard deviations of α and ρ typically exceed standard deviation of τ_DD_ in all strains.

**Figure 9:**
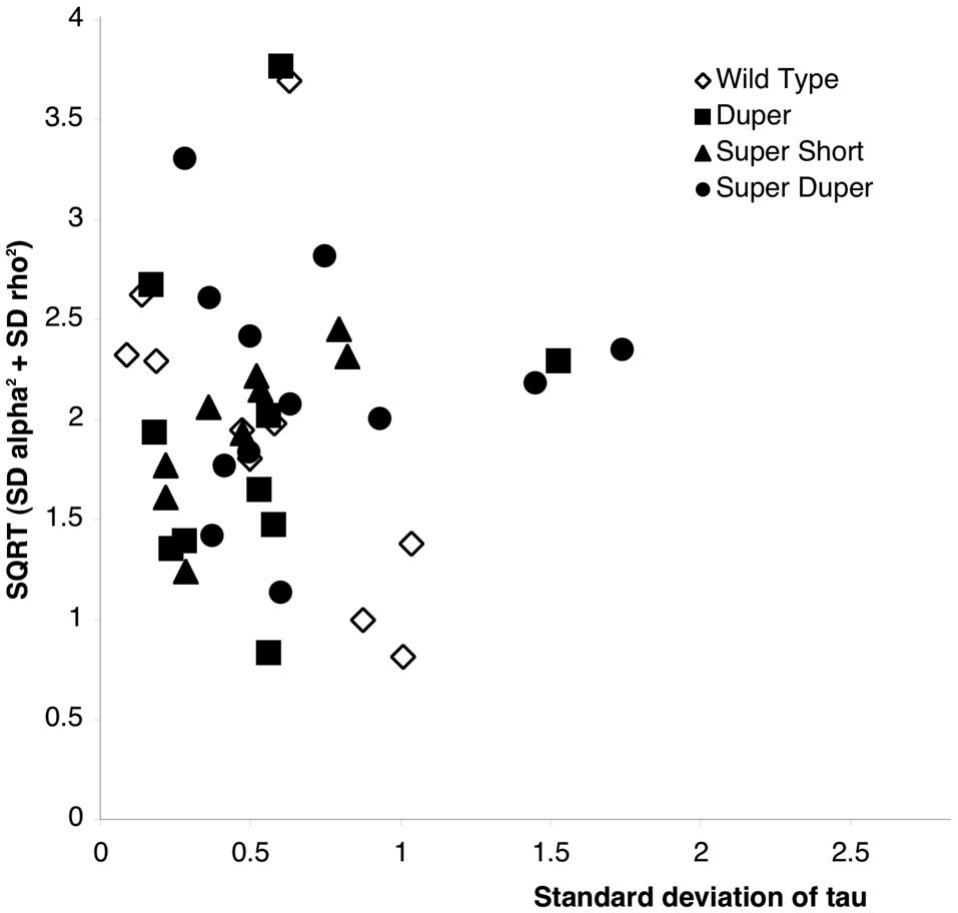
Compensatory changes in variability of α and ρ occur to minimize variability of τ_DD_. Summed variance of activity and rest is greater than the variance of free running period of activity onsets in male TTDD (diamonds), TTdd (squares), ttDD (triangles) and ttdd (circles) hamsters.

I examined whether variability of α or ρ contributed disproportionately to variability of τ_DD_. The standard deviations of α and ρ were generally comparable for animals of a given τ_DD_, and exceeded the variability of τ_onset_ in TTDD, TTdd, ttDD, and ttdd hamsters (Fig. 8). Furthermore, the pooled variation of α and ρ (measured as the square root of the sum of squares of their standard deviations) consistently exceeded the standard deviation of τ_DD_ (Fig. 9). This pattern, which indicates a compensatory balance that offsets fluctuations in the duration of activity and rest in order to conserve period, showed no apparent differences between TTDD, TTdd, ttDD and ttdd hamsters.

## Discussion

The availability of two mutations that shorten circadian period allowed me to examine three related questions: what are the consequences of changes in period length for variability and precision of the circadian oscillator, is period shortening achieved through disproportionate changes in α or ρ, and does period length determine whether activity is related to the preceding or succeeding rest interval?

Circadian biologists have long been impressed by the stability of circadian period, to the point that Pittendrigh and Caldarola [15] proposed that τ is homeostatically defended. Previous studies suggest that minimal variability at a particular value of τ may be a general property of circadian oscillators (see Figure S2). In cyanobacteria, free running period is more variable in strains whose τ is further from 24 h (see Fig. 4 in [16]). Similarly, *frq* and *prd* mutants of *Neurospora crassa* whose period of conidiation differ from the 21.3±0.1h value of the wild type *bd csp-1* strain show a 2- to 4-fold increase in standard error of τ [17]. In contrast, the variability of τ_DD_ in Drosophila appears to be largely invariant over a range of values in both *period* and *timeless* mutants [18]. This question has also been addressed in vertebrates. Aschoff et al. [3] altered the intensity of constant light presented to chaffinches in order to manipulate circadian period, and found that precision is maximal when values of τ are close to 24h. In their classic comparison of four rodent species, Pittendrigh and Daan [2] found that variability of circadian period among hamsters, deer mice, white footed mice, or house mice increases as the species-typical τ_DD_ deviates from 24 h. Although the pattern is striking, its intepretation may be complicated by effects of genetic background indicative of the variety of loci that may influence circadian period [19], [20]. Furthermore, the fact that the hamsters and house mice studied by Pittendrigh and Daan [2] were far more inbred than the *Peromyscus* species may have contributed to their lower inter-individual variability of τ_DD_. Nevertheless, other data gathered within rodent species lend credence to the idea that variability is minimal when τ_DD_ is close to 24 h. Sharma and Chandrashekaran [21] found a significant negative correlation between the standard deviation and the period of activity onsets and offsets in the nocturnal field mouse, *Mus booduga*, with a minimum in mice whose periods are closest to 24 h. Oklejewicz [22] described precision as a U-shaped function of period in wild type and *tau* mutant hamsters, but did not test this trend statistically. Daan and Beersma [23] presented a model in which 2 oscillators whose periods lie on either side of 24 h would generate such a U-shaped function in which precision is related to τ. Data summarized by Takahashi et al. ([20], see Figure S2) allows comparison of τ_DD_ and its standard deviation in a variety of circadian mutants and knockouts as well as their wild type C57/Bl6J controls. The variability shows a striking parabolic trend with a minimum slightly below 24 h. In 7 of the 9 studies cited, mutations or knockouts that alter τ_DD_ induce an increase in the standard deviation, and in one of the exceptions the τ_DD_ of the mutant is closer to 24 h than is that of the wild type control. While the different studies may have included mice bearing different degrees of 129 background, the animals in each study were inbred for multiple generations and the trend is consistent with the pattern reported by Pittendrigh and Daan [2].

Nevertheless, the present data provide little support for the idea that variability of τ_DD_ reaches a minimum at particular values. When closely related hamsters of 8 different genotypes whose mean period ranged from 17.8 to 24.2h were studied in the same laboratory under uniform conditions, there was no statistically significant trend for variability to reach a minimum at the strain-typical τ_DD_. This finding was similar whether cycle or phase variation was used as a measure. Calculation of precision allows the estimate of variation to be normalized for period, and this measure provides similar results whether linear regression or the interval between successive activity onsets is used to estimate variability of period. Only at extremely short endogenous periods – when the duper and *tau* mutations are combined - is precision consistently reduced. It is important to point out that even though the decrement in precision is statistically significant, the free running rhythms of ttdd animals are coherent and typically quite regular (Fig. 3).

Robustness of circadian rhythms may depend upon the existence not only of multiple clock gene orthologs and interlocked and interacting transcriptional feedback loops, but also post-translational processes [4], [5], [24]–[27]. Mutations that differ in their effects on transcriptional vs. post-transcriptional events may have differential or interacting effects on cycle-to-cycle variability. Stabilization of period, resulting in minimal variation, may depend upon a resonance between transcriptional-translational and post-translational events [28]. Mutations that produce a mis-match of time constants (e.g., for rates of turnover of constituent proteins that must interact) might be expected to decrease precision as they alter τ. Like *tau*, the duper mutation may influence post-translational processing, but it could also alter either known core clock components or a critical gene that has not yet been identified. Ultimately, discovery of the locus of the duper mutation will be necessary to understand its effects on precision as well as period, and its interaction with the *tau* mutation. While it seems likely that a single mutation in a core clock component has affected both the period and the PRC, the genetic basis of the mutation remains to be determined. Hopefully, the recessive nature of the duper mutation and the advent of next generation sequencing will soon make this possible.

Although the stability of oscillations may be influenced by effects of mutations on core loops, changes in driven systems that exert feedback effects on the pacemaker may also contribute. In this context, alterations in *dbp* expression within the SCN of ttdd hamsters [11] may be relevant to changes in circadian precision. Furthermore, mutations may affect precision through an influence on intercellular coupling within the pacemaker [29], as well as through changes in cell-autonomous function. It is expected that not only the size but also the strength of interaction within a network of oscillators will influence the stability of the period of the population [29]–[34]. Karatsoreos et al. [35] found that precision, which they defined as deviation from the projected locomotor onset time, was decreased as total activity and α declined and τ_DD_ increased in gonadectomized mice. The colocalization of androgen receptor protein with GRP in the SCN suggested to these authors that a change in function of a selected group of cells which function to coordinate rhythmic function may explain the reduced precision of androgen-deprived mice. No evidence has been gathered on differential effects of either the *tau* or the duper mutation on particular cell types of the hamster SCN. Examination of this possibility awaits development of tools to evaluate appropriate measures of intercellular coupling in wild type and mutant animals.

In seeking an ultimate explanation of relationships between circadian period and precision, Pittendrigh and Daan [2] reasoned that selection would tolerate deviations of circadian period at values of τ_DD_ further from 24 h because these periods ensure more stable phase angles of entrainment. In contrast, variability of period when τ_DD_ is close to 24 h would be more deleterious as it would precipitate dramatic changes in the entrained phase angle. The timing of daily events critical to survival ranging from photosynthesis to arousal and foraging would deviate from the optimal in plants and animals experiencing cycle-to-cycle variation in circadian period. In addition, proper function of mechanisms that insure estrous cyclicity, navigational orientation through use of celestial cues, and measurement of photoperiod to insure appropriate seaonal timing of flowering, diapause, hibernation, reproduction would be adversely affected. These ultimate considerations do not apply to laboratory studies, in which animals are free of pressures that would presumably select against mutations that severely shorten circadian period. To the extent that mutations affect subordinate oscillators, however, increases in cycle or phase variation could be relevant for entrainment of slaves to the pacemaker within the organism. An increase in the cycle variability could alter the coherence of cellular circadian oscillators in peripheral organs, which may damp more rapidly due to the absence of coupling [31]. Mutations that affect precision of peripheral oscillators may also be deleterious if they introduce variability of phase angle relative to the pacemaker as neural and/or hormonal signals dictate that they advance or delay.

Each of the strains studied here here has a short endogenous period, so that all need to entrain to T24 by large phase delays. To the extent that the slope of the delay portion of their PRCs is similar, increased cycle and phase variation would be expected to have equivalent adverse effects on the ability of each of these mutants to maintain a consistent phase angle. The consequences for entrainment of circadian mutations are not predictable, however, solely on the basis of their effects on τ_DD_. The *tau* and duper mutations have different effects on locomotor rhythms in L:D cycles: TTdd entrain more stably to T24 than do TtDD animals even though their periods are similar [11]. Although entrainment of ttdd hamsters is less stable than TTdd or TTDD hamsters, it is less disrupted than that of ttDD animals even though the period is 2 h shorter and circadian precision is significantly reduced [11]. Any deleterious effects of the duper mutation arising from either a reduction in τ_DD_ or an increase in its variability might be mitigated by a change in PRC amplitude which increases both the stability and the range of entrainment.

Some of the differences between genotypes in τ_DD_ may be due at least in part to aftereffects of these varying patterns and degrees of prior entrainment [2]. An influence of such aftereffects on free running rhythms of mutants that are compared in studies such as this is unavoidable unless animals are born and raised in DD. We previously reported the suprising observation that τ_DD_ of a group of duper mutants of both sexes that were never exposed to light was nearly 1 h *shorter* than that of comparable hamsters reared in 14L:10D [10]. The mean precision (τ_DD_/sd) of these 7 animals averaged 57.01±24.7, and the mean phase variation was 0.44±.03. Although raising animals in these conditions and assessing their circadian function presents challenges in husbandry, more extensive and systematic studies that include both wild types and a variety of mutant strains would be necessary to assess the contribution of aftereffects of entrainment to strain differences in circadian precision.

Previous studies suggest that the effect of the duper mutation to produce a Type 0 PRC is accounted for by reduction in the amplitude of the oscillation [11].The present findings indicate that any such change in amplitude is not accompanied by a decrease in the precision of the oscillation. Although stochastic fluctuations in parameters – rate constants or concentrations of molecules that act as feedback signals – might cause greater phase deviation in a lower amplitude cycle, they need not alter stability of period. Thus lability of phase in response to a zeitgeber is not necessarily correlated with susceptability to perturbation as a result of noise. Furthermore, the three measures of the amplitude of the rhythm of locomotor output – the FFT, the autocorrelation coefficient, and the periodogram – were each correlated with the free running period across the range of genotypes studied here. Thus these measures were similar between TTdd and wild type hamsters. If it is true that the effect of the duper mutation on the PRC reflects a decrease in the amplitude of the limit cycle oscillation, this suggests that the amplitude of the oscillator is dissociated from the amplitude of its output.

Constancy of τ might be achieved if deviations in one phase of the oscillation (corresponding to a particular set of molecular events) are compensated by offsetting changes in the duration of another set of steps. I found a consistent negative correlation between α and ρin all hamster strains, such that one phase lengthens as the other shortens in order to achieve conservation of τ (Fig. 4). The present data thus confirm and extend the findings of Aschoff et al. [3] that the durations of α and ρ are negatively correlated in birds and humans. As pointed out by Aschoff, this pattern is counterintuitive on energetic grounds, as one might expect increased energy expenditure incurred during a long α to lead to a longer rest interval so that restorative processes can occur. Perhaps changes in sleep efficiency or more intense recuperative metabolic processes allow replenishment of energy stores in a shorter ρ so that period length can be conserved [36].

Whatever mechanism governs this compensatory mechanism is preserved as τ_DD_ changes and persists even in double mutant (ttdd) hamsters. Although my data confirm the observation that ρ is consistently more strongly correlated with the preceding than the succeeding α, comparisons between hamster mutants do not support Aschoff’s prediction (see Fig. 11 in [3]) that the strength of this relationship depends upon τ (Fig. 7). Aschoff’s finding that variability of α and ρ exceed the variability of τ_onset_, presumably as part of this compensatory mechanism, was apparent in all genotypes (Figs. 8 and 9).

A separate but related question is whether lighting conditions or mutations that alter period exert a disproportionate influence on any particular phase of the cycle. Aschoff et al. [3] presented evidence that lengthening of τ in chaffinches exposed to increased light intensity is correlated with a decrease in the α/ρ ratio. They suggested that the change in τ is mostly due to an increase in the duration of rest in each cycle. Aside from the possibility that this trend might be species-specific, it may be peculiar to effects of light, as it was not evident in people living in temporal isolation in the presence or absence of electric fields. In their study of effects of the *tau* mutation on clock gene expression, Dey et al. [37] observed an increase in the rate of clearance of nuclear PER2 in the SCN. This effect, which is consistent with subsequent findings on the effects of the *tau* mutation on stability of PER2 [9] led these authors to conclude that the mutation selectively shortens the duration of the early subjective night. Contrary to the prediction, I found a proportionate reduction of both α and ρ in bothduper and *tau* mutant hamsters (Fig. 5). Division of the circadian cycle into α and ρmay provide insufficient resolution to identify events that occur during a small portion of the subjective night or day that may be affected by the mutation. Aschoff et al. ( [3], Fig. 7) modeled the relationship between the overt activity rhythm and the underlying oscillator. They postulated that α and ρreflect the crossing of a threshold as the oscillation progresses. Their model predicted that periods of the onset, peak, and offset of activity depend upon the waveform of the oscillation, which may be sinusoidal or skewed. Variability in the threshold of activation of activity (“vertical noise”) is thought to interact with variability in the basic oscillation (“horizontal noise”). Such variations, which would be both expected to change the values of α and ρ, may correspond to changes in wakeup time vs. oscillator amplitude and may be affected by *tau* and duper mutations in different ways. Assessment of the waveform of the pacemaker may be achieved by electrophysiological techniques, and such studies indicate that the SCN oscillation is surprisingly sinusioidal [38]. Measurement of multiple unit activity of the SCN may reflect the output of the oscillator as much as pacemaker function itself, however; in order to assess the amplitude of the circadian oscillator it will likely be necessary to achieve a complete description of the excursions of expression of all of the core clock genes. Accomplishment of this task not only in wild types but also in mutants will allow us to understand how τ, α, ρ, and the range of entrainment are determined.

As previously noted [13], [22] the cycle-to-cycle variability of period was greater in females than in males when either activity onset or acrophase was used as a phase marker. Only in the shortest period (ttdd) females was variability of activity offset greater than in males. It is speculated that during proestrus, the advanced phase of activity onset (along with the increase amount of activity) increases the likelihood of encounters with fertile males [39], [40]. Ovulation takes place near the middle of the subjective night, however, so that even at the offset of activity the female is still likely to be fertile. Effects of ovarian hormones on circadian function differ from those of the mutations studied here: increases in α and the number of wheel revolutions per cycle that occur when serum concentrations of estradiol are high, as on the nights of proestrus and estrus, are correlated with a *decrease* in τ_DD_. The effects of ovariectomy and sex steroid replacement upon the periods of activity onset, acrophase and offset, as well as their variability, merit further study.

In conclusion, the duper mutant provides a new tool with which to study the mechanisms of circadian rhythmicity, the formal properties of these oscillations, and their physiological impact. Neither mutation selectively alters α or ρ. Interactions between duper and *tau* remain to be understood. Nevertheless, the present data show that the changes in τ that are produced by these mutations, alone or in combination, do not markedly compromise the precision of the circadian pacemaker.

## Supporting Information

Figure S1 **Precision in individual male (A)TTDD, (B)TTdd, (C)ttDD and (D)ttdd hamsters plotted relative to τ_DD_.** Within genotypes, precision did not reach a minimum at the mean circadian period and neither linear nor quadratic regression trends were statistically significant.
(TIF)

Figure S2 **Previous studies in a variety of species have tested whether variability of circadian period is minimal at a particular value of τ_DD_.** This figure plots data reported in (A) bioluminescent reporter expression in *Synechococcu*s (data of Kondo et al. 1994; see [16]); (B) conidiation in *Neurospora crassa* (data of Lakin-Thomas and Brody, 1985; see [17]); (C) eclosion in *Drosophila melanogaster* (*Per* and *Timeless* mutants, data of Rothefluh et al., 2000, see [18]); and (D) locomotor activity rhythms of mice (data compiled by Takahashi et al., 2008; see [20], supplement 1). Variability between mutants cannot be evaluated statistically as the data are drawn from a variety of studies in which conditions differed.
(TIF)
