# Supplementary figures and images for: Does the Precision of a Biological Clock Depend upon Its Period? Effects of the Duper and tau Mutations in Syrian Hamsters
Source: PLoS One. 2012 May 15;7(5):e36119. doi: 10.1371/journal.pone.0036119 (PMC3352912; doi:10.1371/journal.pone.0036119)

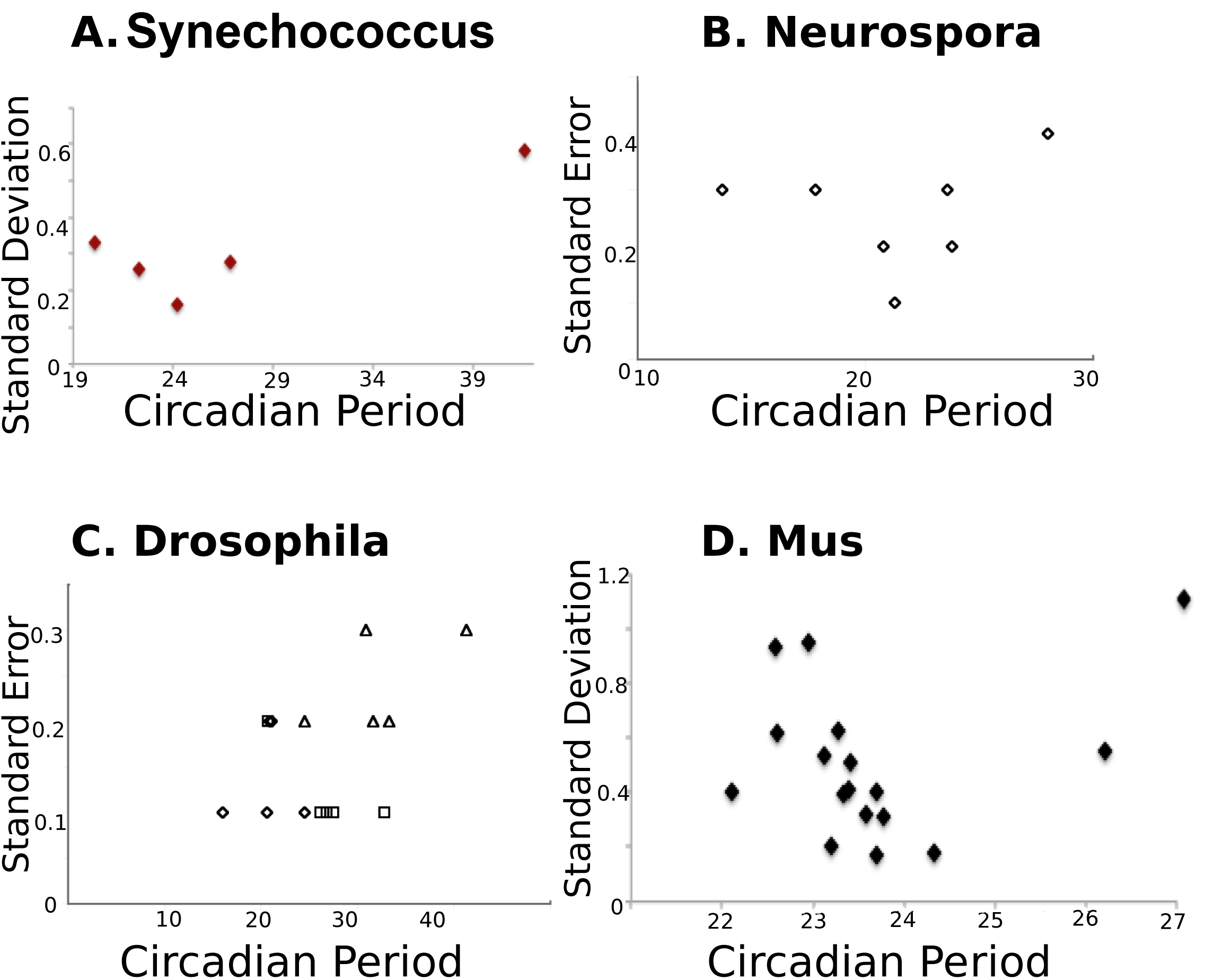

Supplement: Figure S2 — Previous studies in a variety of species have tested whether variability of circadian period is minimal at a particular value of τDD. This figure plots data reported in (A) bioluminescent reporter expression in Synechococcus (data of Kondo et al. 1994; see [16]); (B) conidiation in Neurospora crassa (data of Lakin-Thomas and Brody, 1985; see [17]); (C) eclosion in Drosophila melanogaster (Per and Timeless mutants, data of Rothefluh et al., 2000, see [18]); and (D) locomotor activity rhythms of mice (data compiled by Takahashi et al., 2008; see [20], supplement 1). Variability between mutants cannot be evaluated statistically as the data are drawn from a variety of studies in which conditions differed. (TIF) [file pone.0036119.s002.tif]
